# Cross-Amplification of *Vicia sativa* subsp. *sativa* Microsatellites across 22 Other *Vicia* Species

**DOI:** 10.3390/molecules20011543

**Published:** 2015-01-16

**Authors:** Sebastin Raveendar, Gi-An Lee, Young-Ah Jeon, Yun Jeong Lee, Jung-Ro Lee, Gyu-Taek Cho, Joon-Hyeong Cho, Jong-Hyun Park, Kyung-Ho Ma, Jong-Wook Chung

**Affiliations:** 1 National Agrobiodiversity Centre, National Academy of Agricultural Science, Rural Development Administration, Jeonju 560-500, Korea; 2 Department of Biological and Environmental Science, Dongguk University, Seoul 100-175, Korea; 3 Food Grain Policy Division, Ministry of Agriculture, Food and Rural Affairs, Sejong-si 339-012, Korea

**Keywords:** cDNA-SSR, genetic diversity, transferability, *Vicia* L.

## Abstract

The temperate and herbaceous genus *Vicia* L. is a member of the legume tribe Fabeae of the subfamily Papilionoideae. The genus *Vicia* comprises 166 annual or perennial species distributed mainly in Europe, Asia, and North America, but also extending to the temperate regions of South America and tropical Africa. The use of simple sequence repeat (SSR) markers for *Vicia* species has not been investigated as extensively as for other crop species. In this study, we assessed the potential for cross-species amplification of cDNA microsatellite markers developed from common vetch (*Vicia sativa* subsp. s*ativa*). For cross-species amplification of the SSRs, amplification was carried out with genomic DNA isolated from two to eight accessions of 22 different *Vicia* species. For individual species or subspecies, the transferability rates ranged from 33% for *V*. *ervilia* to 82% for *V*. *sativa* subsp. *nigra* with an average rate of 52.0%. Because the rate of successful SSR marker amplification generally correlates with genetic distance, these SSR markers are potentially useful for analyzing genetic relationships between or within *Vicia* species.

## 1. Introduction

The genus *Vicia* L. is a member of the legume tribe Fabeae (sometimes referred to as “Vicieae”) of the subfamily Papilionoideae [1]. *Vicia* L. comprises approximately 210 species that are widely distributed in temperate regions of Europe, Asia, and the Americas [2]. *Vicia* species are morphologically diverse and it is rather difficult to account for the entire genetic variation existing in *Vicia* species using morphological characters [3]. Archaeological evidence suggests that the Mediterranean region is the principal center of diversification [4]. Large scale structural changes were observed in *Vicia* chromosomes; thus, cytological studies have been performed to determine chromosome number and morphology [5]. Similarly, karyological studies have been performed for taxonomic classification [6]; however, earlier morphological and cytogenetic studies had limited ability to provide stable taxonomic classifications [7]. Currently, molecular markers are successfully used to identify divergence among wild populations.

In general, a number of molecular markers are applied in crop breeding programs to achieve genome coverage, high reproducibility, and greater polymorphic and genomic distribution with locus specificity, which are considered simple and inexpensive to genotype [8]. However, the development of DNA sequence-based molecular markers is expensive. To overcome this limitation, transferability of molecular markers among species has been studied [9,10,11]. Among the molecular markers, simple sequence repeat (SSR) markers are considered to be efficient due to their co-dominant and multi-allelic nature, relative abundance, high genome coverage, and reproducibility of results [12]. Recently, with the development of transcriptome sequencing, expressed sequence tags (ESTs) have been deposited in Genbank databases (dbEST), and provide information for the potential development of molecular markers [13]. Due to the conserved nature of transcribed regions, EST-based markers are considered to be important because of their high rate of transferability across species [14]. Given the current lack of legume genomic resources, the conservation of genome structure assures transfer from model legume species to other legume species [15]. In legumes, conserved and cross-species transferable markers have been used to describe the relationship between model legume and other legume species [16]. We examined the transferability of SSR microsatellite markers from the *V*. *sativa* genome to distantly related *Vicia* species. Our findings provide information on the rates of cross-species transferability of SSRs, and the relative degree of SSR polymorphism that will allow the establishment of more consistent relationships among the *Vicia* species.

## 2. Results and Discussion

In total, 36 *V*. *sativa* cDNA-derived SSR primer pairs were tested for their ability to cross-amplify across 22 other *Vicia* species. Of the 36 SSR loci assayed, the transferability rate ranged from 33% for *V*. *ervilia* to 82% for *V*. *sativa* subsp. *nigra* (Table 1). The average percentage of transferability for all 22 species was 52%. The highest rate of transferability was from *V*. *sativa* subsp. *nigra* (82%), followed by *V. lutea* (74%). The 36 *V*. *sativa* primers produced a total of 3,168 amplicons of which 1,922 amplicons were polymorphic (Table 1). SSRs are considered to be the markers of choice for genome mapping in crop breeding programs. The high level of transferability of SSRs observed in *Vicia* indicated that the SSR loci were conserved among these related taxa. Conservation of SSR flanking regions has been reported in closely related legumes [17,18,19]. High cross-species transferability rates ranging from 29.4% to 61.7% were observed with SSR markers across the seven legume genera [20]. In this study, the average percentage of cross-species transferability among all 22 species was 52%, which coincides with reported transferability in *Vicia* species [21].

**Table 1:** Rates of successful amplification in *Vicia* species.

Species (Sample Size)	Theoretical Amplicons (No.)	Amplicons (No.)	Transferability (%)
*V. amoena* (4)	144	77	53
*V. anatolica* (2)	72	36	50
*V. articulata* (4)	144	51	35
*V. benghalensis* (4)	144	72	50
*V. cassubica* (2)	72	28	39
*V. costata* (2)	72	25	35
*V. cracca* (4)	144	73	51
*V. disperma* (2)	72	30	42
*V. ervilia* (4)	144	47	33
*V. hirsuta* (2)	72	40	56
*V. hyrcanica* (4)	144	85	59
*V. lutea* (2)	72	53	74
*V. michauxii* (4)	144	84	58
*V. montbretii* (4)	144	90	62
*V. narbonensis* (4)	144	66	46
*V. pannonica* (2)	72	31	43
*V. peregrina* (4)	144	74	51
*V. sativa* subsp. *nigra* (8)	288	236	82
*V. sativa* subsp. *sativa* (14)	504	473	94
*V. tetrasperma* (4)	144	78	54
*V. unijuga* (2)	72	35	49
*V. villosa* (4)	144	95	66
*V. villosa* subsp. *varia* (2)	72	43	60
**Mean**			54

**Figure 1:**
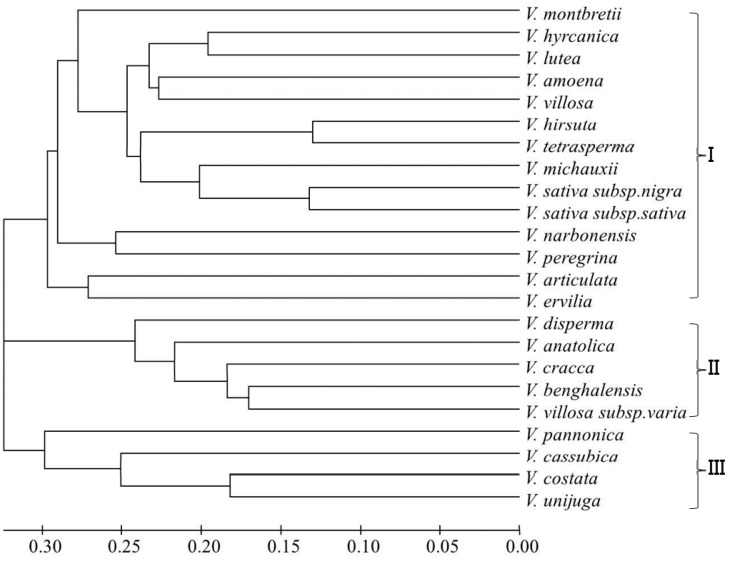
Phylogenetic tree for 23 *Vicia* species based on 36 microsatellite markers.

To determine the genetic diversity structure and relationships between 23 species of *Vicia*, we used polymorphism scores at 36 microsatellite loci (Table 2). The number of alleles (N_A_) per locus varied widely among the markers with values ranging from one to 17 with an average of 6.30 alleles per locus (Table 2). The frequency of major alleles (M_AF_) per locus varied from 0.25 to 0.97 with an average of 0.59. Observed heterozygosity (H_O_) values ranged from 0.00 to 0.69 with an average of 0.12, expected heterozygosity (H_E_) values ranged from 0.00 to 0.90 with an average of 0.50, and overall polymorphism information content (PIC) values ranged from 0.00 to 0.84 with an average of 0.50. Of the 36 markers, GBSSR-VSspS-091 was found to be monomorphic. The test for Hardy-Weinberg equilibrium (HWE) and pairwise linkage disequilibrium (LD) of 35 polymorphic loci were showed significant deviation from HWE (*p* < 0.05), whereas 16 pairwise combinations GBSSR-VSspS (020 and 023, 023 and 073, 073 and 076, 119 and 140, 126 and 166, 181 and 203, 203 and 247, 247 and 301, 301 and 310, 310 and 313, 313 and 067, 305 and 088, 088 and 308, 308 and 024, 024 and 028, 028 and 075) were non-significantly deviated from LD (*p* < 0.05). The HWE suggesting that these significant deviations possibly resulted from the presence of null alleles (Table S1 and Table S2). Using the microsatellite markers, we further characterized the genetic diversity among the *Vicia* accessions. Unweighted pair group method with arithmetic mean (UPGMA) cluster analysis was performed using Powermaker v.3.25 software [22]. The cluster analysis revealed that the genotypes of all of the *Vicia* species could be differentiated clearly and grouped into three main clusters (Figure 1). The UPGMA dendrogram derived from the 36 SSR loci showed 3 clusters in that *V*. *pannonica*, *V*. *cassubica*, *V*. *costata*, and *V*. *unijuga* grouped in one clade. *V*. *disperma*, *V*. *anatolica*, *V*. *cracca*, *V*. *benghalensis*, and *V*. *villosa* subsp. *varia* were distinguishable from other *Vicia* species, with *V*. *benghalensis* and *V*. *villosa* subsp. *varia* exhibiting the greatest similarity to each other. The remaining 14 *Vicia* species, including *V*. *sativa* subsp. *sativa*, were dispersed with mixed levels of similarity (Figure 1). However, the dendrogram did not indicate any clear division among the *Vicia* accessions. Chung *et al.* [23] reported a cluster analysis of *V*. *sativa* subsp. *sativa* in which no clear clustering pattern of geographically close accessions was observed, indicating that the association between genetic similarity and geographical distance was of low significance.

Microsatellite transferability among related species arises from the presence of conserved homologous DNA flanking regions, but successful cross-species amplification is inversely related to the evolutionary distance of the species [24]. Typically, closely related species with conserved SSR priming sites yield high frequencies of cross-species amplification. The transfer of SSR primers is occasionally possible from more distantly related species. Our findings provide evidence for the potential transferability of SSRs across 23 *Vicia* species. The high frequency of cross-transferability of SSR from *V. sativa* subsp. *sativa* to 22 other species examined in this study may be due to their close relatedness, as all of the genera belong to the subfamily Papilionoideae. More recently, Akash *et al.* [21] reported the cross-transferability of EST-SSRs to eight *Vicia* species, also members of the same subfamily Papilionoideae. In this study, the number of alleles detected at each locus ranged from one to 17, with an average of 6.3 alleles. The number of alleles observed for each primer in this study was lower than that reported for *Vicia* by Akash *et al.* [21]. The PIC values for the 36 SSR primers varied from 0.12 for GBSSR-VSspS-067 to 0.84 for GBSSR-VSspS-179 (Table 2). The average PIC value was 0.50, indicating that these loci were highly informative and polymorphic. The genetic relatedness we observed among the 23 *Vicia* species was not previously reported. In this study, 36 SSR markers showed satisfactory cross-amplification within the expected allele size range across 23 *Vicia* species (Table 1). This result indicates that this set of loci could be used in population genetics studies addressing species cohesion and delimitation, phylogeography, and barriers to gene flow for other *Vicia* species.

**Table 2:** Characteristics of 36 microsatellite markers in *Vicia* spp.

Marker	N_A_	M_AF_	H_O_	H_E_	PIC
GBSSR-VSspS-020	3	0.83	0.00	0.30	0.26
GBSSR-VSspS-023	4	0.42	0.09	0.70	0.63
GBSSR-VSspS-073	5	0.52	0.19	0.70	0.60
GBSSR-VSspS-076	4	0.66	0.11	0.50	0.43
GBSSR-VSspS-080	5	0.47	0.11	0.70	0.63
GBSSR-VSspS-079	6	0.63	0.05	0.60	0.53
GBSSR-VSspS-090	9	0.30	0.06	0.80	0.79
GBSSR-VSspS-099	6	0.47	0.13	0.70	0.62
GBSSR-VSspS-107	10	0.50	0.23	0.70	0.64
GBSSR-VSspS-102	5	0.52	0.10	0.60	0.56
GBSSR-VSspS-125	6	0.50	0.13	0.70	0.66
GBSSR-VSspS-119	6	0.64	0.17	0.50	0.45
GBSSR-VSspS-140	3	0.48	0.00	0.60	0.56
GBSSR-VSspS-126	3	0.50	0.00	0.60	0.55
GBSSR-VSspS-166	2	0.72	0.00	0.40	0.32
GBSSR-VSspS-179	12	0.27	0.16	0.90	0.84
GBSSR-VSspS-181	9	0.61	0.35	0.60	0.57
GBSSR-VSspS-203	3	0.61	0.09	0.50	0.45
GBSSR-VSspS-247	3	0.82	0.00	0.30	0.28
GBSSR-VSspS-301	4	0.67	0.00	0.50	0.45
GBSSR-VSspS-310	7	0.54	0.36	0.60	0.60
GBSSR-VSspS-313	2	0.97	0.00	0.10	0.06
GBSSR-VSspS-067	4	0.94	0.03	0.10	0.12
GBSSR-VSspS-251	20	0.46	0.69	0.80	0.74
GBSSR-VSspS-037	11	0.37	0.25	0.80	0.77
GBSSR-VSspS-268	4	0.53	0.11	0.60	0.56
GBSSR-VSspS-042	7	0.49	0.10	0.70	0.67
GBSSR-VSspS-305	10	0.44	0.13	0.70	0.60
GBSSR-VSspS-088	2	0.76	0.08	0.40	0.30
GBSSR-VSspS-308	6	0.91	0.05	0.20	0.17
GBSSR-VSspS-024	3	0.90	0.00	0.20	0.18
GBSSR-VSspS-028	2	0.90	0.07	0.20	0.16
GBSSR-VSspS-075	6	0.38	0.08	0.70	0.68
GBSSR-VSspS-091	1	1.00	0.00	0.00	0.00
GBSSR-VSspS-115	17	0.35	0.27	0.80	0.82
GBSSR-VSspS-129	16	0.25	0.21	0.90	0.83
**Mean**	6.3	0.59	0.12	0.50	0.50

N_A_, number of alleles; M_AF_, major allele frequency; H_O_, observed heterozygosity; H_E_, expected heterozygosity; PIC, polymorphism information content.

## 3. Experimental Section

### 3.1. Plant Materials

The *Vicia* species used in this study (88 accessions of 23 species) were collected from the National Agrobiodiversity Center (http://www.genebank.go.kr/), Rural Development Administration, Republic of Korea (Table S1). For genomic comparisons, *Vicia* species, including *V*. *sativa* subsp. s*ativa*, seeds, were germinated and leaf samples were collected when the plants were 3 weeks old. All tissue samples were preserved at −80 °C until analyses were performed.

### 3.2. Amplification of SSRs

A total of 36 SSR primers developed and assayed previously for *V*. *sativa* subsp. *sativa* [23] were used to determine whether SSR primers were transferable to other *Vicia* genomes (Table 2). Genomic DNA was extracted from *Vicia* samples using a DNeasy^®^ Plant Mini kit (Qiagen, Valencia, CA, USA) according to the manufacturer’s instructions. Fresh leaf tissue from each accession was used for each extraction and was ground well in liquid nitrogen. DNA was resuspended in 100 μL water, and dilutions were made to 10 ng/μL followed by storage at either −20 °C or −80 °C. Genomic DNA was quantified using a Nanodrop/UVS-99 instrument (ACTGene, Piscataway, NJ, USA), and the A260/A280 nm ratio was determined. DNA quality was confirmed on a 0.8% agarose gel. Randomly selected SSR primer pairs were validated experimentally, and forward primers were synthesized with the addition of M13 sequence to enable fluorescent tail addition during the PCR amplification process [25]. PCR conditions included a hot-start at 95 °C for 10 min, followed by 10 cycles at 94 °C for 30 s, 60–50 °C for 30 s, and 72 °C for 30 s, followed by 25 cycles at 94 °C for 30 s, 50 °C for 30 s, 72 °C for 30 s, and a final elongation step at 72 °C for 10 min. PCR products were separated and visualized using a QIAxcel Gel Electrophoresis System (Qiagen). SSR alleles were resolved on an ABI 3130xl Genetic Analyzer (Applied Biosystems, Foster City, CA, USA), and sized precisely at the base pair level based on an internal size standard (35–500 bp, GeneScan 500ROX; Applied Biosystems).

### 3.3. Statistical Analysis

To characterize genetic variation, the observed N_A_, M_AF_, observed H_O_, expected H_E_, and PIC were calculated using PowerMarker [22] and GenAlEx (version 6.5) software [26]. Cluster analysis of the 23 species was performed using the unweighted pair group method with arithmetic mean (UPGMA) phylogenetic cluster analysis, and unrooted tree construction was based on the CS chord 1967 distance method in the PowerMarker version 3.25 software.

## 4. Conclusions

The high levels of cross-species transferability observed in this study indicate that the *V*. *sativa* cDNA-derived SSRs primer pairs described here may be suitable for assessments of genetic diversity and population structure, construction of high-density linkage maps, and conservation and molecular marker-assisted breeding of target species. We estimated the intra-specific diversity of widely distributed *Vicia* species based on the representative material (88 accessions of 23 species). The results of this initial study could help in the generation of tools to identify genetic distributions of *Vicia*, and may potentially provide a feasible means to understand the genomic synteny with other legumes.

## Supplementary Materials

Supplementary materials can be accessed at: http://www.mdpi.com/1420-3049/20/01/1543/s1.
